# Physio-Biochemical, Anatomical, and Molecular Analysis of Resistant and Susceptible Wheat Cultivars Infected with TTKSK, TTKST, and TTTSK Novel *Puccinia graminis* Races

**DOI:** 10.3390/plants13071045

**Published:** 2024-04-08

**Authors:** Hayat Ali Alafari, Yaser Hafez, Reda Omara, Rasha Murad, Khaled Abdelaal, Kotb Attia, Amr Khedr

**Affiliations:** 1 Department of Biology, College of Science, Princess Nourah bint Abdulrahman University, P.O. Box 84428, Riyadh 11671, Saudi Arabia; 2 EPCRS Excellence Center, Plant Pathology and Biotechnology Laboratory, Agricultural Botany Department, Faculty of Agriculture, Kafrelsheikh University, Kafr El-Sheikh 33516, Egyptdrrashaymurad55@gmail.com (R.M.);; 3 Wheat Diseases Research Department, Plant Pathology Research Institute, Agricultural Research Center, Giza 12619, Egypt; 4 Center of Excellence in Biotechnology Research, King Saud University, P.O. Box 2455, Riyadh 11451, Saudi Arabia; kattia1.c@ksu.edu.sa

**Keywords:** stem rust, Sr genes, plant fungal interaction, wheat, molecular analysis, anatomical structure

## Abstract

Stem rust, caused by *Puccinia graminis* f.sp. *tritici*, is one of the most dangerous rust diseases on wheat. Through physiological, biochemical, and molecular analysis, the relationship between the change in resistance of 15 wheat cultivars to stem rust disease and the response of 41 stem rust resistance genes (*Sr,s*) as well as TTKSK, TTKST, and TTTSK races was explained. Some cultivars and Sr genes, such as Gemmeiza-9, Gemmeiza-11, Sids-13, Sakha-94, Misr-1, Misr-2, Sr31, and Sr38, became susceptible to infection. Other new cultivars include Mir-3 and Sakha-95, and Sr genes *13*, *37*, *40*, *GT*, and FR*2/SRTT3-SRTT3-SR10 remain resistant. Some resistance genes have been identified in these resistant cultivars: *Sr2*, *Sr13*, *Sr24*, *Sr36*, and *Sr40*. *Sr31* was not detected in any cultivars. Reactive oxygen species such as hydrogen peroxide and superoxide, enzymes activities (catalase, peroxidase, and polyphenoloxidase), and electrolyte leakage were increased in the highly susceptible cultivars, while they decreased in the resistant ones. Anatomical characteristics such as the thickness of the epidermis, ground tissue, phloem tissue and vascular bundle diameter in the midrib were decreased in susceptible cultivars compared with resistant cultivars. Our results indicated that some races (TTKSK, TTKST, and TTTSK) appeared for the first time in Egypt and many other countries, which broke the resistant cultivars. The wheat rust breeding program must rely on land races and pyramiding genes in order to develop new resistance genes that will survive for a very long time.

## 1. Introduction

Wheat is one of the most significant crops in the world for human food and animal feed [1,2]; therefore, many efforts are made to increase its grain production in Egypt. Egypt’s total wheat production is approximately 9 million tons, with consumption at 20 million tons. There are many challenges facing researchers to increase crop production under various environmental conditions such as water deficit [3,4], salinity [5], and rust diseases [6,7,8,9]. Stem rust is one of the most dangerous diseases and is caused by the fungus *Puccinia graminis* f. sp. *tritici* (Pgt) [10,11]. Under ideal environmental situations, the fungus can generate new physiological races that target resistant cultivars and propagate epidemically, resulting in up to 100 percent yield losses across wide areas during epidemic years. The most efficient, cost-efficient, and ecologically benign method of managing this disease is the identification and development of resistant genotypes [12]. Planting resistant cultivars is advised since resistant host plants are the most efficient way to prevent the rust disease. The production of wheat by both small and big scale farmers is currently threatened by the recent re-emergence of a highly virulent race, generally known as Ug99 and designated as TTKSK based on North American nomenclature [13,14,15].

The availability of “green bridges” and a sizable quantity of airborne urediniospores that start early epidemics are both guaranteed by the staggered planting strategy. The current commercial wheat cultivars are extremely susceptible to the new race because susceptible cultivars are constantly under attack from the rust pathogen [16]. Therefore, achieving long-lasting resistance to wheat stem rust necessitates ongoing pathogen characterization, as well as the discovery and use of new resistance genes that outcompete the dominant pathogenic races. The traditional technique for identifying resistance genes that are probably present in crop cultivars is gene postulation. Therefore, the definition of resistance genes was based on molecular markers [17]. It is based on the principle of gene-for-gene specificity, in which the infection types produced by pathogen isolates on cultivars under research are compared to infection types produced by the same isolates on near-isogenic lines bearing a single known resistance gene [18]. The genetic makeup of wheat cultivars in Egypt’s main wheat-growing areas indicate that major genes, minor genes, or both combined control disease resistance; nevertheless, the complementing effects of major genes may improve a variety’s response and result in higher degrees of resistance. For the introduction of new efficient resistance genes into released cultivars, knowledge of the stem rust resistance genes in those cultivars is crucial. Environmental stress factors are associated with the formation of ROS, which causes lipid peroxidation under any abiotic and biotic stresses conditions [19,20,21,22]. The increase of hydrogen peroxide (H_2_O_2_) and super oxide (O_2_) under infection was observed. Enzymatic and non-enzymatic components such as Catalase (CAT), ascorbate peroxidase (APX), peroxidase (POX), polyphenol oxidase (PPO), ascorbic acid, glutathione, carotenoids, and proline are very important in plants’ defense system against stress conditions [23,24]. CAT, POX, and PPO up-regulation play a critical role in protecting plants from pathogen attack and scavenge ROS as well as alterations in membrane permeability as a result of either biotic or abiotic stresses [25,26]. Therefore, the purpose of this work was to use physiological, chemical, genetic, and anatomical analysis to determine cultivars’ sensitivity and resistance to stem rust disease, as well as the genes responsible for resistance, related to the new physiological races.

## 2. Results

### 2.1. Evaluation of Wheat Cultivars against Stem Rust at the Adult Stage

**Figure 1:**
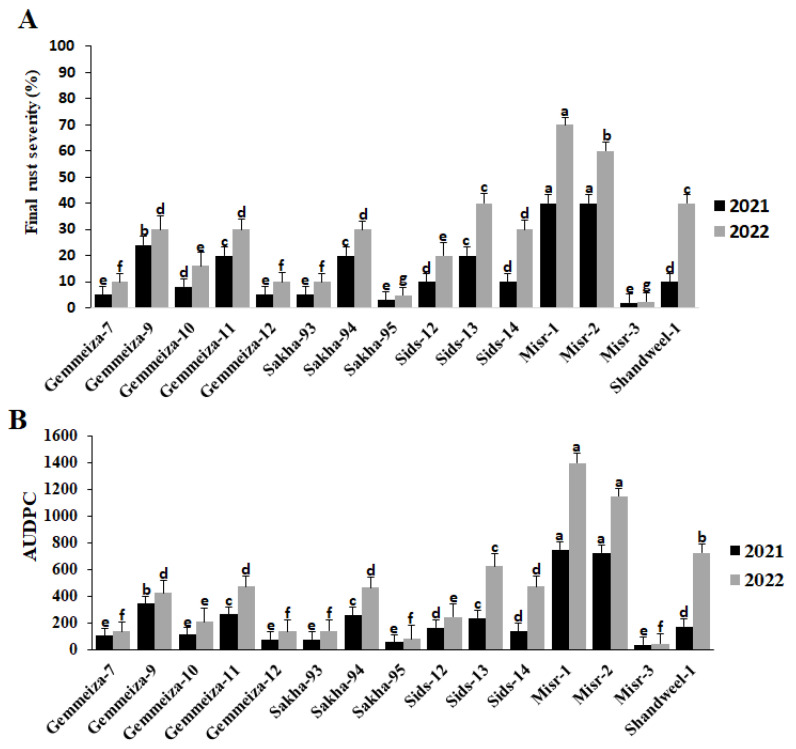
Final rust severity (**A**) and AUDPC (**B**) of 15 wheat cultivars against stem rust during the 2021 and 2022 seasons. The different letters indicate the presence of significance between them.

Fifteen wheat cultivars were evaluated against stem rust under field conditions in the Kafrelsheikh governorate, Egypt, in both seasons 2021 and 2022 (Figure 1). As a result of the cultivars’ reaction to the disease, the cultivars with the highest final disease severity were Misr-1 and Misr-2, followed by Gemmeiza-9, Gemmeiza-11, Sids-13, and Sakha-94 (Figure 1A). Also, these cultivars recorded the highest values for AUDPC during the two seasons (Figure 1B). In the seasons 2021 and 2022, the Sakha-95 and Misr-3 cultivars had the lowest final disease severity and AUDPC values (Figure 1A,B). In presenting these results, it was found that the disease severity and AUDPC in the second season were higher than in the first season, which prompts us to try to explain these results.

### 2.2. Evaluation of 41 Stem Rust Resistance Genes (Sr,s)

Forty-one stem rust resistance genes (*Sr,s*) were evaluated for stem rust under field conditions during the seasons of 2021 and 2022 (Table 1 and Table 2). The most resistant genes in 2021 were *13*, *22*, *26*, *28*, *35*, *37*, *40*, *GT*, *Brigardier* (*Sr31*), *PBW343* (*Sr31*), *Sisson* (*Sr31+36*), *Chris* (*Sr7a*, *Sr12*, *Sr6*), and FR*2/SRTT3-SRTT3, *SR10*, and in 2022 they were *13*, *37*, *40*, *GT*, and FR*2/SRTT3-SRTT3, *SR10*, which achieved zero disease severity (Table 2). Through these results, it was found that there are five genes (*13*, *37*, *40*, *GT*, and FR*2/SRTT3-SRTT3-SR10) that had complete resistance to this disease during both seasons, and the second season was higher in disease severity than the first season.

### 2.3. Identification of Stem Rust Resistance Genes in Wheat Cultivars

**Figure 2:**
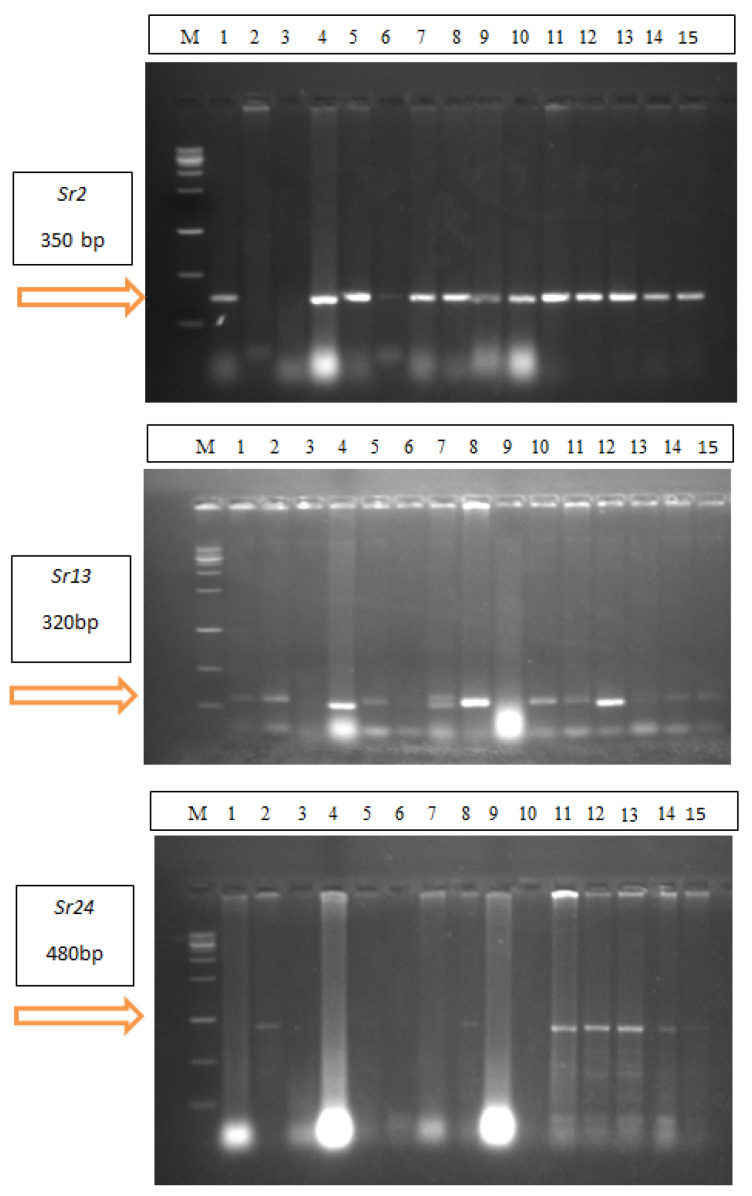
Detection of Sr2, Sr13, and Sr24 genes in 15 wheat cultivars (M = Ladder, 1 = Gemmeiza-9, 2 = Gemmeiza-12, 3 = Sakha-94, 4 = Sakha-95, 5 = Sids-14, 6 = Sids13, 7 = Sids-12, 8 = Misr-3, 9= Gemmeiza-7, 10 = Sakha-93, 11 = Misr-1, 12 = Gemmeiza-10, 13 = Gemmeiza-11, 14 = Misr-2 and 15 = Shandweel-1).

**Figure 3:**
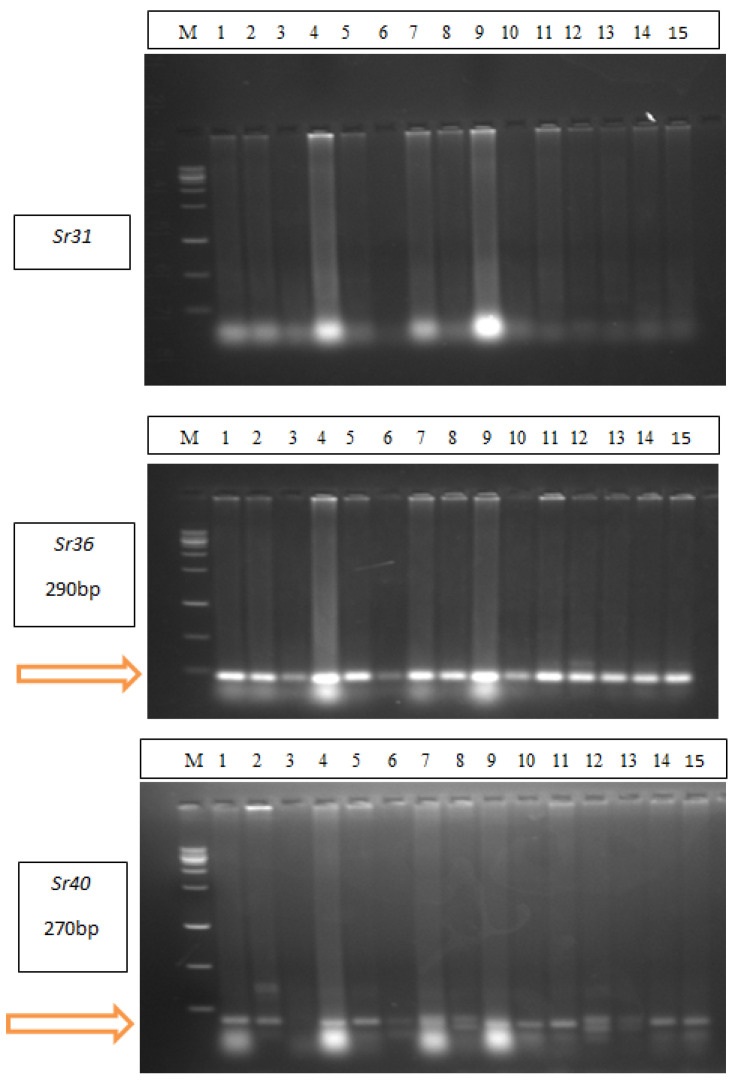
Detection of Sr31, Sr36, and Sr40 genes in 15 wheat cultivars (M = Ladder, 1 = Gemmeiza-9, 2 = Gemmeiza-12, 3 = Sakha-94, 4 = Sakha-95, 5 = Sids-14, 6 = Sids13, 7 = Sids-12, 8 = Misr-3, 9 = Gemmeiza-7, 10 = Sakha-93, 11 = Misr-1, 12 = Gemmeiza-10, 13 = Gemmeiza-11, 14 = Misr-2 and 15 = Shandweel-1).

To explain the resistance of wheat cultivars to stem rust, it was necessary to identify the stem rust resistance genes (*Sr,s*) responsible for resistance in 15 wheat cultivars. Therefore, the most resistant genes such as *Sr2*, *Sr13*, *Sr24*, *Sr31*, *Sr36*, and *Sr40* were selected and defined in 15 wheat cultivars (Figure 2 and Figure 3). Gene *Sr2* was detected in 13 wheat cultivars at 350bp. except the Gemmeiza-12, Sakha-94, and Sids-13 cultivars. Meanwhile, *Sr13* was identified in four wheat cultivars, Sakha-95, Misr-3, Sakh-93, and Gemmeiza-10, at 320bp. Gene *Sr24* was detected at 480bp. in Gemmeiza-12, Misr-3, Misr-1, Misr-2, Gemmeiza-10, and Gemmeiza-11 (Figure 2). *Sr40* was detected in nine wheat cultivars, Gemmeiza-9, Gemmeiza-12, Sakha-95, Sids-14, Gemmeiza-7, Sakha-93, Misr-1, Misr-2, and Shandweel-1, at 270bp. Gene *Sr36* was identified in all wheat cultivars at 290 bp. On the other hand, *Sr31* was not detected in any cultivars (Figure 3).

### 2.4. Effect of Stem Rust Infection on Enzyme Activity in Different Wheat Cultivars

**Figure 4:**
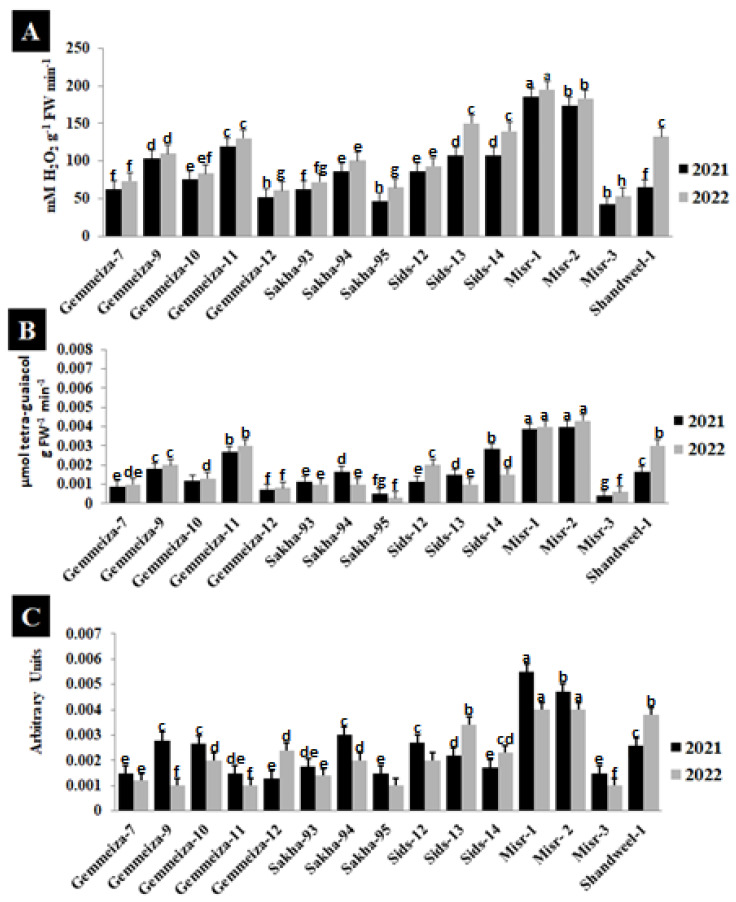
Catalase (**A**), peroxidase (**B**), and polyphenol oxidase (**C**) activities in some cultivars infected with stem rust in the 2021 and 2022 seasons. The different letters indicate the presence of significance between them.

The activity of catalase, peroxidase, and polyphenol oxidase in 15 wheat cultivars infected with stem rust was studied to analyze the resistance and susceptibility to this disease in the two seasons (Figure 4). An increase in the catalase, peroxidase, and polyphenol oxidase activity was observed in the highly susceptible cultivars (Misr-1 and Misr-2), and a decrease in their activity was seen in the highly resistant cultivars (Sakha-95 and Misr-3) (Figure 4A–C).

### 2.5. Effect of Stem Rust on Electrolyte Leakage, Chlorophyll a, and Chlorophyll b of Different Cultivars

**Figure 5:**
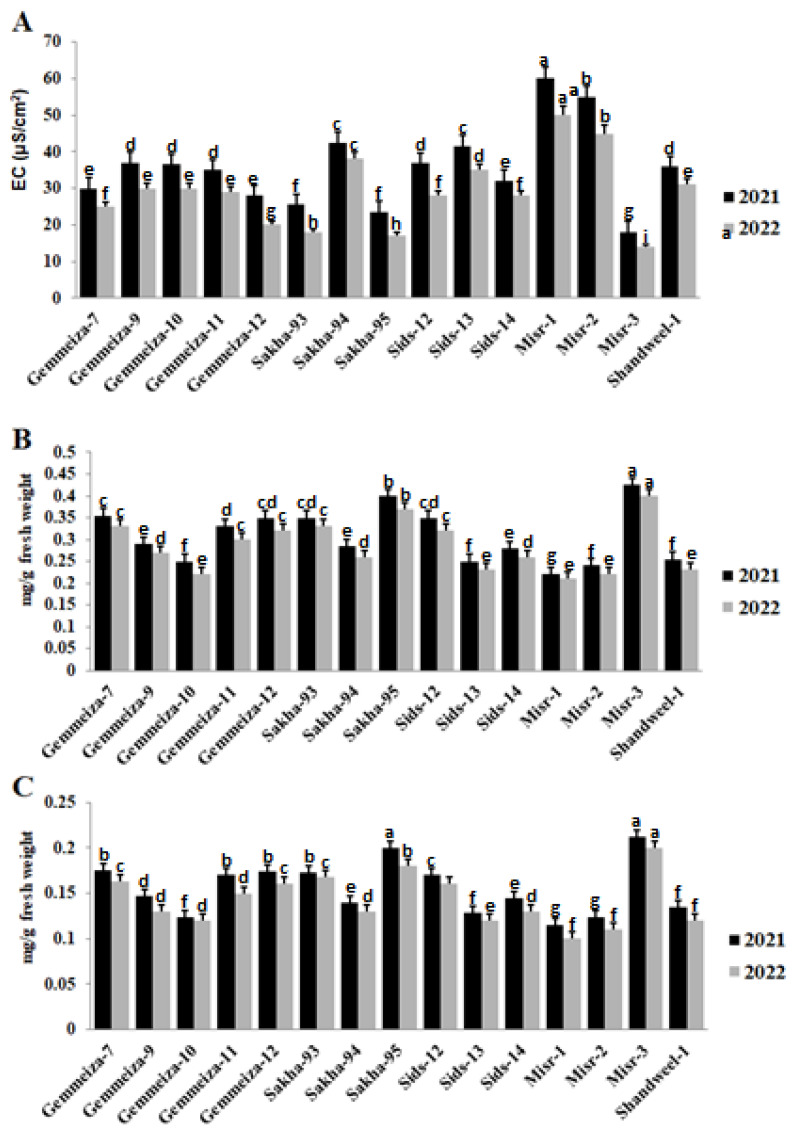
Electrolyte leakage (**A**), chlorophyll a (**B**) and chlorophyll b (**C**) in some wheat cultivars infected with stem rust in the 2021 and 2022 seasons. The different letters indicate the presence of significance between them.

Also, the results of infection with stem rust on the wheat cultivars under study had a clear effect on the level of electrolyte leakage, chlorophyll a, and chlorophyll b in the 2021 and 2022 seasons (Figure 5). It was clear that the electrolyte leakage level increased in the highly susceptible cultivars (Misr-1 and Misr-2) and decreased in the resistant ones (Sakha-95 and Misr-3) (Figure 5A). On the contrary, it was observed that the level of chlorophyll a and chlorophyll b increased in the resistant cultivars (Sakha-95 and Misr-3) and was reduced in the susceptible ones (Misr-1 and Misr-2) (Figure 5B,C).

### 2.6. Effect of Stem Rust on Reactive Oxygen Species (ROS) of Four Wheat Cultivars

**Figure 6:**
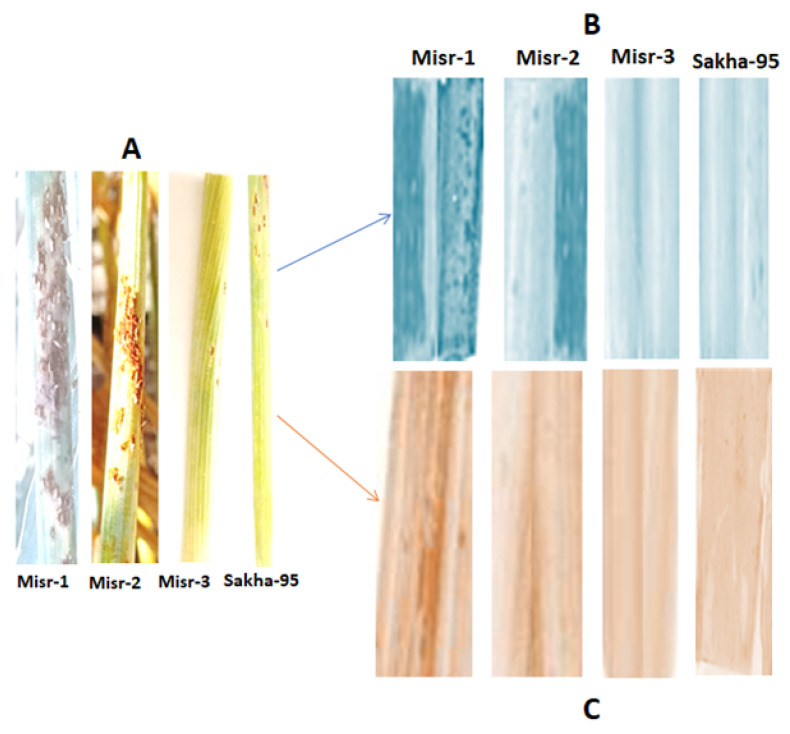
Symptoms of stem rust (**A**), purple discoloration (**B**) and brown discoloration (**C**) of superoxide and hydrogen peroxide, respectively, in Misr-1, Misr-2, Misr-3, and Sakha-95 cultivars. Sakha 95 and Misr 3 are resistant to the disease that were used to compare with the more susceptible varieties Misr 1 and Misr 2, so they are similar.

Our results in Figure 6 show that superoxide and hydrogen peroxide as purple and brown discoloration, respectively, were measured in four cultivars Misr-1, Misr-2, Misr-3, and Sakha-95. In resistant cultivars, namely Misr-3 and Sakha-95, the discoloration decreased compared with the susceptible cultivars Misr-1 and Misr-2 (Figure 6).

### 2.7. Effect of P. graminis f. sp. tritici on the Anatomical Characters of Four Wheat Cultivars

**Figure 7:**
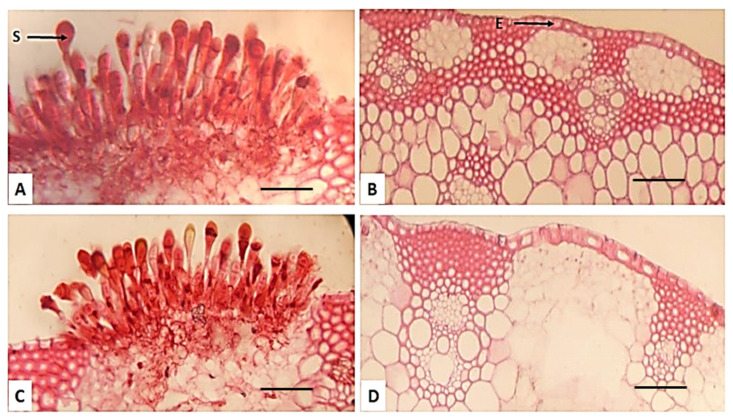
Transverse sections of wheat cultivars’ stems ((**A**) Misr-1, (**B**) Misr-3, (**C**) Misr-2, and (**D**) Sakha-95), inoculated with *Puccinia graminis* in the 2021/2022 season. (Magnification ×100). E: Epidermis, S: spores.

According to our results, Figure 7 shows that the fungus *P. graminis* f. sp. tritici decreased most anatomical characteristics in susceptible cultivars, such as the thickness of the epidermis and the thickness of ground tissue and phloem tissue, as well as the diameter of the vascular bundle in the midrib in susceptible cultivars (Misr-1 and Misr-2) compared with resistant cultivars (Misr-3 and Sakha-95).

### 2.8. Distribution of P. graminis f. sp. tritici Races in Egypt and Other Countries

**Figure 8:**
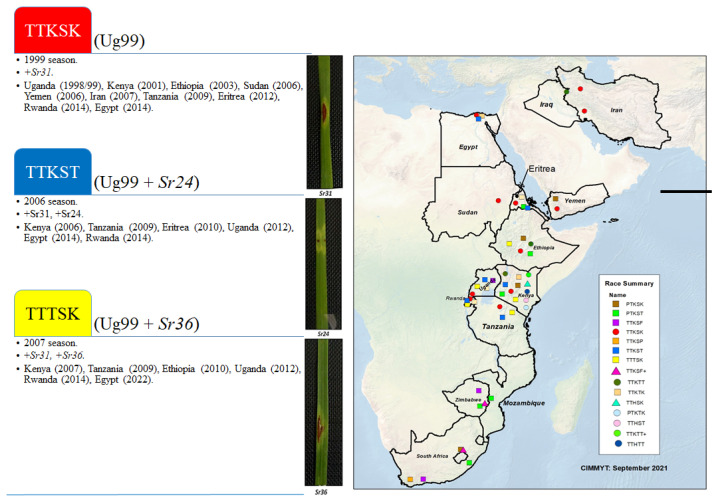
Distribution of TTKSK, TTKST, and TTTSK races in Egypt and other countries, as well as their symptoms on some stem rust resistance genes.

The researchers attempted to interpret the prior findings in order to explain why some wheat cultivars behaved differently when infected with the stem rust fungus. It was noted that various races (TTKSK, TTKST, and TTTSK) that were defined in Egypt and many other nations from 1999 to 2022 debuted for the first time (Figure 8). TTKSK appeared in Uganda (1998/9), Kenya (2001), Ethiopia (2003), Iran (2007), Tanzania (2009), Eritrea (2012), Rwanda (2014), and Egypt (2014). TTKST appeared in Kenya (2006), Tanzania (2009), Egypt (2014), Rwanda (2014), and TTTSK appeared in Kenya (2007), Ethiopia (2010), Uganda (2012), Rwanda (2014), and Egypt (2022). For the first time, these races were able to disrupt the *Sr31*, *Sr24*, and *Sr36* resistance genes (Figure 8).

## 3. Discussion

Stem rust is one of the most damaging diseases to wheat. Most cultivars were susceptible to this disease, which was the main disease in the first half of the 20th century (1930s–1940s), until a source of resistance was imported from Kenya. Giza-139 was the first cultivar to be resistant to stem rust; it was introduced in 1947. Semi-dwarf cultivars and CIMMYT-introduced lines contained various resistance genes in the early 1970s. But now, it has been found that some wheat cultivars are resistant to stem rust disease, such as Gemmeiza-10, Sids-13, and Gemmeiza-11, while the Sids-12, Shandweel-1, Misr-1, and Misr-2 cultivars became susceptible [27]. As a result of the emergence of the Ug99 race in Uganda, all Egyptian wheat varieties were evaluated against this race, all of which were susceptible to infection. Therefore, Misr-1 and Misr-2 cultivars were selected from the CIMMYT wheat genotypes and evaluated in Uganda, Kenya, and Ethiopia, and they proved their resistance to stem rust disease, especially for this race. Then the two varieties were cultivated in these countries to overcome this race (Ug99). In the two growing seasons, fifteen wheat cultivars were evaluated against stem rust in Egypt. The Misr-1, Misr-2, Gemmeiza-9, Gemmeiza-11, Sids-13, and Sakha-94 cultivars recorded the highest final disease severity and the highest values of area under disease progress. As the two cultivars, Misr-1 and Misr-2, become susceptible to stem rust disease, therefore, it was necessary to introduce new resistant cultivars instead of those susceptible to infection, and these cultivars were Misr-3 (from CIMMYT wheat genotypes) and Sakha-95 [28]. In this study, they showed the highest resistance to stem rust. The change in the behavior of wheat cultivars towards resistance to stem rust made us think about evaluating the 41 stem rust resistance genes (*Sr,s*) responsible for resistance to this disease in the 2021 and 2022 seasons. Through these results, it was found that there are five genes (*13*, *37*, *40*, *GT*, and FR*2/SRTT3-SRTT3-SR10) that have complete resistance to this disease during the two seasons, and that the second season was higher in disease severity than the first season [29]. Previous studies were conducted to evaluate the gene expression under stem rust infection [30,31,32,33,34]. It is possible to link the breaking of gene resistance to the change in the behavior of wheat cultivars to the disease. These genes were identified in the cultivars through molecular markers. It was noticed that some genes such as *Sr2* and *Sr24* were known in wheat cultivars such as Misr-1 and Misr-2. These genes were more effective against this disease. *Sr2* is considered to be a slow-rusting gene or form of adult plant resistance (APR) [35,36]. It was noticed in numerous Kenyan varieties, including Kenya Plume and CIMMYT varieties, Pavon 76, Juchi 2000, and Kritati. *Sr24* is widely used in wheat breeding programs worldwide. Since then, it has been introgressed into many wheat genotypes [36]. The *Sr24* gene was ineffective for some variants in the lineage of Ug99, but it is effective for the new races: TKTTF, TTTTF, and many *P. graminis* races in China [37]. But after the emergence of Ug99 (TTTKS) in Uganda (1998/99), Kenya (2001), Ethiopia (2003), Sudan (2006), Yemen (2006), Iran (2007), Eritrea (2012), Rwanda (2014), and Egypt (2014), the resistance of most cultivars broke, especially *Sr31* [38,39,40]. Then the TTKST race appeared and broke the resistance gene *Sr24* in Kenya (2006), Tanzania (2009), Eritrea (2010), Uganda (2012), Rwanda (2014), and Egypt (2014). Also, the resistance gene *Sr36* was broken by the TTTSK race in Kenya (2007), Tanzania (2009), Ethiopia (2010), Uganda (2012), Rwanda (2014), and Egypt (2022) [41]. This race (TTTSK) was recorded for the first time in Egypt, which prompts us to explain the change in the behavior of some cultivars in resistance to stem rust disease, and especially that this gene (*Sr36*) was detected in all cultivars under study. On the contrary, the result of gene evaluation in the field indicated the efficiency of *Sr13* in 2021 and 2022, and it was determined in the resistant cultivars: Misr-3 and Sakha-95.

Another point is that two crucial roles for the ROS are played during infection defense. The first one is the buildup of ROS, which prevents or kills infections while supporting hypersensitive necrosis [22]. The second function is the reduction of ROS levels, which promotes the activation of antioxidants and the expression of resistance genes in tissues close to infection sites [22]. Specifically, ROS and H_2_O_2_ serve a dual role by inducing the overexpression of resistance genes, encouraging localized host and pathogen cell death, and promoting antioxidant activities. The plants’ typical defensive system against ROS accumulation is an antioxidant defense system that is formed by antioxidant enzymes like CAT, PPO, and POX. Following inoculation, these enzymes were upregulated in the susceptible cultivars, whereas CAT and POX activities were noticeably elevated in the resistant cultivars. Under several stressors, these enzymes play a vital role in reducing ROS levels or scavenging to detoxify and remove their harmful effects [42,43,44,45,46]. Also, the increase in electrolyte leakage may be due to the effect of the pathogen on the cell membrane in the susceptible cultivars (Misr-1 and Misr-2) and its permeability, whereas the membrane permeability of the resistant cultivars (Misr-3 and Sakha-95) were not affected under infection. The variation in anatomical characteristics such as the epidermis, xylem, and phloem tissues in resistant and susceptible wheat cultivars was studied, and the increase in stem anatomical characteristics was observed in the resistant cultivars. The detrimental effects of *P. graminis* f. sp. *tritici* on cell division, elongation, and enzyme activity, as well as the various growth characteristics of wheat plants, led to the decline in anatomical characteristics in susceptible cultivars [47]. This result is in accordance with the findings of some researchers [48,49]. According to the results, Egypt was considered one of the countries at risk for the spread of Ug99, especially given that most wheat genotypes in Egypt were based on CIMMYT germplasm. Therefore, one must rely on land races and pyramiding genes in the wheat rust breeding program in order to obtain new resistance genes that will last for long periods of time.

## 4. Materials and Methods

The current study was performed during the 2021 and 2022 seasons at the experimental farms of the Faculty of Agriculture, Kafrelsheikh University, Kafr El-Sheikh, Egypt. The laboratory experiments were conducted at Excellence Center and Plant Pathology & Biotechnology Lab, Department of Agricultural Botany, Faculty of Agriculture, Kafrelsheikh University, Egypt.

### 4.1. Field Experiments

#### Evaluation of Some Wheat Cultivars and Stem Rust Resistance Genes (*Sr,s*) at the Adult Stage

In the Kafr El-Shakh governorate, 15 cultivars (Table 2) and 41 stem rust resistance genes (*Sr,s*) (Table 3) were tested under field conditions during the 2021 and 2022 seasons. The complete randomized block design with three replicates was used in these experiments, and there were three rows in the experimental unit (3 m long and 30 cm apart and 5g seed rate for each row). The experiment was encircled by a 1.5 m belt and a 1 m ditch that demarcated entries that were susceptible to stem rust, such as “Morocco”. The spreader was artificially inoculated using TTKSK, TTKST, and TTTSK races of *P. graminis* during late tillering and late elongation stages. The inoculation was done by shaking or brushing rusted material over the plant leaves to create an initial film of free water on the plants, which is necessary for spore germination and for the infection to take hold. According to the approach used by Peterson et al. [50], disease severity (DS) was measured four times, every 10 days, during the two consecutive seasons as the percentage of leaves covered in rust pustules. Rust reaction was expressed in five types [51], i.e., immune = (0), resistant = (R), moderately resistant = (MR), moderately susceptible = (MS), and susceptible = (S). Then rust reaction was transformed to average coefficient of infection (ACI) values according to the methods adopted by Saari and Wilcoxson [52]. According to an equation proposed by Pandey et al. [53], AUDPC was calculated in the tested cultivars.

### 4.2. Laboratory Experiments

#### 4.2.1. Molecular Markers

A total of 15 wheat cultivars were examined to identify the six stem rust resistance genes (*Sr2*, *Sr13*, *Sr24*, *Sr31*, *Sr36* and *Sr40*) by using 6 specific primers purchased from SBS Company, Jinjiang City, China. Molecular analysis was carried out at the EPCRS Excellence Center, Faculty of Agriculture, Kafer El-sheikh University, Egypt.

The extraction of DNA was carried out using a modified procedure that was based on Dellaporta et al.’s [54] methodology. The PCR Amplification procedure followed these instructions. Six specific primers were used to detect the six stem rust resistance genes as shown in Table 4. The PCR reaction mixture (15μL) contained 5 ng DNA template, 10 pmol of forward primer, 10 pmol of reverse primer, 0.1 U of Taq DNA polymerase (Bioline GmbH, Luckenwalde, Germany), 25 mM of MgCl2, 2 mM dNTPs, and 10× PCR buffer in 96 well thermal cyclers (Applied Biosystem Thermal Cycler, Singapore). The reaction conditions were as follows: initial denaturation was for 5 min at 94◦C, followed by the initial 37 cycles of denaturation for 1 min at 94◦C, annealing (Table 4), and extension at 72 for 2 min. Subsequently, a 10 min final extension at 72◦C was done. PCR products of SSR markers were checked for amplification on 2% agarose gel. All the PCR amplification bands were separated using the electrophoresis method on 2% agarose gels prepared in 1× TBE buffer stained with ethidium bromide. The Mid-Range DNA Ladder 100bp-3kbp linear sale (Jena Bioscience, Jena, Germany) was used to detect the molecular weight of the tested samples. The Mid-Range DNA Ladder 100bp-3kbp linear sale (Jena Bioscience, Jena, Germany) was used to detect the molecular weight of the tested samples.

#### 4.2.2. Determination of O_2_- and H_2_O_2_

O_2_- and H_2_O_2_ were shown to be purple in NBT and reddish-brown in DAB staining, respectively. Vacuum infiltrated stems were treated with 0.1 *w*/*v* percent NBT (Sigma-Aldrich, Steinheim, Germany) or 0.1 *w*/*v* percent DAB (Fluka, Buchs, Switzerland). NBT- and DAB-treated samples were cleaned in 0.15 *w*/*v* percent trichloroacetic acid in ethanol: chloroform 4:1 *v*/*v* for 1 day after being exposed to sunlight for 20 min and 2 h, respectively [61]. The samples were washed with water and placed in 50% glycerol prior to analysis. Utilizing nicked eyes or ChemiImager 4000 digital imaging equipment, the discoloration of stems was recorded.

#### 4.2.3. Assays of Antioxidant Enzymes

For enzyme assays in plants, 0.5 g wheat stem material was homogenized at 0–4 °C in 3 mL of 50 mM TRIS buffer (pH 7.8) containing 1 mM EDTA-Na2 and 7.5% polyvinylpyrrolidone. The samples were centrifuged for 20 min at 4 °C (12,000 rpm), and then the total soluble enzyme activity was measured in the supernatant using a spectrophotometer. All measurements were done at 25 °C, using a spectrophotometer. The enzyme assays were tested three times. CAT activity was assayed according to Aebi [62] using a spectrophotometer. Changes in absorbance at 240 nm were recorded for 3 min every 30 sec intervals. PPO activity was determined according to the method of Malik and Singh [63]. The absorbance was recorded at 495 nm for 3 min. The activity was stated as the increase in absorbance min^−^^1^ g^−^^1^ fresh weight. POX activity was recorded for the crude extract according to Hammerschmidt et al. [64], and the changes in absorbance were measured at 470 nm every 3 min. Enzyme activity was recorded as the increase in absorbance (min^−^^1^ g^−^^1^ fresh weight).

#### 4.2.4. Electrolyte Leakage (EL%)

EL% was assayed according to Szalai et al. [65] and Whitlow et al. [66]. Twenty discs (1 cm^2^) of leaves were placed into individual vials with 25 mL deionized water (Milli-Q 50, Millipore, Bedford, MA, USA). Flasks were shaken for 20 h at ambient temperature to facilitate electrolyte leakage from injured tissues. Initial electrical conductivity was recorded for each vial using an Acromet AR20 electrical conductivity meter. Flasks were then immersed in a hot water bath at 80 °C for 1 h to encourage cell rupture. The vials were placed on the Innova 2100 platform shaker for 20 h at 21 °C, final conductivity was recorded for each flask. EL was recorded for each bud as follows: initial conductivity/final conductivity × 100.

#### 4.2.5. Chlorophyll a and b Content

The concentration of chlorophyll (Chl) of one gram of fresh leaves was extracted with 5 mL N,N-dimethyl-formamid overnight at 5 °C, then Chl. a and b were estimated using a spectrophotometer at 663 and 647 nm as mg/g f w [67]. The concentrations were calculated with the following equations:

Chl. A = 12.76_A663_ − 2.79_A647_ (mg/g fresh weight).

Chl. B = 20.76 _A647_ − 4.62_A663_ (mg/g fresh weight).

#### 4.2.6. Anatomical Studies

During the second growing season, stem samples (1 cm length) 70 days old were collected from the middle of the fourth internode from the apex. The samples underwent killing and fixation (F.A.A.) and were rinsed in 50% ethyl alcohol before being dehydrated in a typical butyl alcohol series. The specimens were then heated to between 56 and 58 degrees in paraffin wax. A rotary microtome type 820 was used to cut transverse sections that were 12 micrometres thick. The sections were mounted in Canada balsam, fixed with albumin, and dyed with safranin [68,69]. The sections were examined using a light microscope and photographed.

#### 4.2.7. Statistical Analysis

The data were analyzed using SPSS software for Windows version 25.0. All comparisons were assessed through a one-way analysis of variance (ANOVA) test. To identify significant differences among treatment means, Duncan’s multiple range test was employed at a significance level of *p* ≤ 0.05.

## 5. Conclusions

Gemmeiza-9, Gemmeiza-11, Sids-13, and Sakha-94 cultivars became susceptible, while new cultivars, Sakha-95 and Misr-3, were resistant. Physiological, biochemical, and molecular analysis differentiated between them. By defining the stem rust resistance genes such as *Sr2*, *Sr13*, *Sr24*, *Sr31*, *Sr36*, and *Sr40*, it became clear that there is a great similarity in the genes defined within these cultivars, which shows the similarity in pedigree. Also, these cultivars were obtained from the same source (CIMMYT). These cultivars’ resistance was broken as a result of novel races such as TTKSK, TTKST, and TTTSK, which appeared for the first time in Egypt and other countries. The wheat rust breeding program must rely on land races and pyramiding genes in order to develop new resistance genes that will survive for a very long time.

## Figures and Tables

**Table 1:** Final rust severity of stem rust resistance genes (*Sr,s*) in the Kafrelsheikh governorate during 2021 and 2022 seasons.

NO.	Sr Gene	Kafrelsheikh Governorate	
		2021	2022
**1**	*2*	5.63	6.00
**2**	*5*	73.33	76.67
**3**	*6*	36.67	93.33
**4**	*7b*	53.33	66.67
**5**	*8a*	63.33	83.33
**6**	*9a*	46.67	76.67
**7**	*9b*	43.33	66.67
**8**	*9e*	63.33	86.67
**9**	*9g*	43.33	86.67
**10**	*9d*	66.67	76.67
**11**	*10*	63.33	66.67
**12**	*11*	36.67	46.67
**13**	*13*	0.00	0.00
**14**	*17*	2.66	6.00
**15**	*17*	76.67	46.67
**16**	*21*	66.67	83.33
**17**	*22*	0.00	5.00
**18**	*24*	1.33	26.00
**19**	*26*	0.00	5.00
**20**	*28*	0.00	7.66
**21**	*30*	66.67	36.67
**22**	*31*	2.33	18.67
**23**	*31*	2.66	4.33
**24**	*31*	2.133	1.33
**25**	*31*	0.00	2.33
**26**	*31*	1.13	2.8
**27**	*31*	0.00	2.00
**28**	*35*	0.00	6.00
**29**	*36*	1.133	9.333
**30**	*37*	0.00	0.00
**31**	*38*	2.43	4.33
**32**	*40*	0.00	0.00
**33**	*Tmp*	76.67	76.67
**34**	*GT*	0.00	0.00
**35**	*MCN*	66.67	70.00
**Combined genes**			
**36**	*24+31*	1.06	9.00
**37**	*36+31*	0.00	7.66
**38**	*Sr36*, *6*	8.33	3.66
**39**	*Sr6*, *24*, *36*, *1RS-Am*	0.73	1.46
**40**	*Sr7a*, *Sr12*, *Sr6*	0.00	1.06
**41**	*SRTT3*, *SR10*	0.00	0.00

**Table 2:** The pedigree list of the fifteen cultivars used in this study.

No.	Wheat Cultivar	Pedigree
1	Sakha-93	SAKHA92/TR810328 S.8871-1S-2S-1S-0S.
2	Sakha-94	OPATA/RAYON//KAUZ. CMBW90Y3180-OTOPM-3Y-010M-010M-010Y-10M-015Y-0Y-0AP-0S.
3	Sakha-95	PASTOR//SITE/MO/3/CHEN/AEGILOPS SQUARROSA(TAUS)//BCN/4/WBLL1CMSA01Y00158S-040P0Y-040M030ZTM-040SY-26M-0Y-0SY-0S
4	Gemmeiza-7	CMH74A.630/5X//SERI82/3/AGENT. GM4611-2GM-3GM-1GM-0GM.
5	Gemmeiza-9	ALD”S”/HUAS//CMH74A.630/SX.GCM4583-5GM-1GM-0GM.
6	Gemmeiza-10	MAYA74”S”/ON / 1160- 147 /3/ BB/ G11/4/ CHAT “S”/5/ CROW “S”GCM 5820- 3GM- 1GM- 2GM- 0GM.
7	Gemmeiza-11	BOW”S” /KVZ”S”// 7C/SERI82/3/GIZA168 /SKHA61. GM7892-2GM-1GM-2GM-1GM-0GM.
8	Gemmeiza-12	OTUS/3/SARA/THB//VEE. CCMSS97Y00227S-5Y-010M-010Y -010M-2Y-1M-0Y-0GM
9	Sids-12	BUC//7C/ALD/5/MAYA74/ON//1160147/3/BB/GLL/4/CHAT”S”/6/MAYA/VUL//CMH74A.630//4*SX. SD7096-4SD-1SD-1SD-0SD.
10	Sids-13	AMAZ19=KAUZ”S”//TSI/SNB”S”. ICW94-0375-4AP-2AP-030AP-0APS-3AP-0APS-050AP-0AP-0SD.
11	Sids-14	SW8488*2/KUKUNACGSS01Y00081T-099M-099Y-099M-099B-9Y-0B-0SD
12	Misr-1	OASIS/SKAUZ//4*BCN/3/2*PASTOR.CMSSOYO1881T-050M-030YO3OM-30WGY-33M-0Y-0S
13	Misr-2	SKAUZ/BAV92. CMSS96M0361S-1M-010SY-010M-010SY-8M -0Y-0S
14	Misr-3	ATTILA*2/PBW65*2//KACHU CMSS06Y00582T-099TOPM-099Y-099ZTM-099Y-099M-10WGY-0B-0EGY
15	Shandweel-1	SITE/MO/4/NAC/TH.AC//3*PVN/3/MIRLO/BUC CMSS93B00567S-72Y-010M-010Y-010M-3Y-0M-0THY-0SH

**Table 3:** The pedigree list of the forty-one stem rust resistance genes (*Sr,s*) used in this study.

No.	Sr Gene	Tester	No.	Sr Gene	Tester
1	*2*	CnS(Hope3B)	22	*31*	Sr31 (Benno)/6*LMPG-6 DK42
2	*5*	ISr5-Ra	23	*31*	Kavkaz
3	*6*	ISr6-Ra	24	*31*	Federation 4/Kavkaz
4	*7b*	ISr7b-Ra	25	*31*	Brigardier
5	*8a*	ISr8a-Ra	26	*31*	Clement
6	*9a*	ISr9a-Ra	27	*31*	PBW343
7	*9b*	W2691Sr9b	28	*35*	W3763-SR35
8	*9e*	Vernstein	29	*36*	W2691SrTt-1
9	*9d*	ISr9d-Ra	30	*37*	W2691 SR37TT2
10	*9g*	CnsSr 9g	31	*38*	VPM-1
11	*10*	W2691Sr10	32	*40*	RL 6087 Dyck
12	*11*	ISr11-Ra	33	*Tmp*	CnsSr Tmp
13	*13*	W2691SR13	34	*GT*	BT-SrGt
14	*17*	Combination	35	*MCN*	McNair 701
15	*17*	LC/KENYA HUNTER-SR17	36	*24+31*	Siouxland
16	*21*	CnS_T_monococcum	37	*36+31*	Sisson
17	*22*	SWSR22T.B.	38	*Sr36*, *6*	Roughrider
18	*24*	LcSr24Ag	39	*Sr6*, *24*, *36*, *1RS-Am*	Fleming
19	*26*	EAGLE-SR26,SR9G	40	*Sr7a*, *Sr12*, *Sr6*	Chris
20	*28*	W2691 SR28KT	41	*SRTT3*, *SR10*	FR*2/SRTT3-SRTT3, SR10
21	*30*	BtSr30Wst			

**Table 4:** Primer, sequences, Annealing Temperature and references from Sr genes associated markers.

Genes		Primer Sequence	Annealing Temp. (°C)	Amplicon Size (bp)	Reference
***Sr2***	F R	CAA GGG TTG CTA GGA TTG GAA AAC AGA TAA CTC TTA TGA TCT TAC ATT TTT CTG	55 °C	350 bp	[55]
***Sr13***	F R	CGGAGCAAGGACGATAGG CACCACACCAATCAGGAACC	54 °C	320 bp	[56]
***Sr24***	F R	CAC CCG TGA CAT GCT CGT A AAC AGG AAA TGA GCA ACG ATG T	58 °C	480 bp	[57]
***Sr31***	F R	CTCTGTGGATAGTTACTTGATCGA CCTAGAACATGCATGGCTGTTACA	55 °C	1200 bp	[58]
***Sr36***	F R	CGT CGA AAA CCG TAC ACT CTC C GCG AAA CAG AAT AGC CCT GAT G	61 °C	290 bp	[59]
***Sr40***	F R	CAAGGAAATAGGCGGTAACT ATTTGAGTCTGAAGTTTGCA	51 °C	270 bp	[60]

## Data Availability

Data are contained within the article.
